# Reasons Why the Office of Coroner Should Be Held by a Member of the Medical Profession

**Published:** 1892-06

**Authors:** 


*Reasons why the Office of Coroner should be Held by a Member of the Medical Profession.* By FREDERICK W. LOWNDES. Pp. 34. London: J. & A. CHURCHILL.

The reasons given in this recently-written but undated pamphlet are sound, and if read by those who have the power of election, will no doubt influence their votes in future contests for the office of Coroner. The public at last seem to be taking more interest in the matter of Coroners and their duties, as there are at present two Bills before Parliament proposing to alter "The Coroners' Act of 1887."

"How to Avoid a Hasty Election" will be settled by the Bill (if passed) which was introduced in the Commons by Sir A. Rollit on March 30th last.

The author has inadvertently fallen into error by saying that the newly-elected Coroner for Birmingham is required to devote the whole of his time to the duties of the office.

In a foot-note of page 8, we must read for "Coroners' Act of 1887," "Municipal Corporations' Act, 1882," and remember that the existing law is that the Coroner's deputy in the large cities must be either a barrister or solicitor; it is important therefore that such alteration be at once made in the "Municipal Corporations' Act, 1882," as will enable a medical man to act as deputy to the Coroner in municipal corporations and cities.

If the "Coroners' Act Amendment Bill," introduced by Sir Walter Foster on February 19th, becomes law, it will be all the more important for the Coroner to have a sound knowledge of *post mortem* appearances in health and disease, as by this Bill he will be the only person required to view the body.

The several quotations from Dr. B. W. Richardson's speeches are very strong arguments in favour of medical Coroners.

